# Editorial to “Associations of the fibrosis‐4 index with left atrial low‐voltage areas and arrhythmia recurrence after catheter ablation: Cardio‐hepatic interaction in patients with atrial fibrillation”: Fibrosis is a cause or an outcome!

**DOI:** 10.1002/joa3.13084

**Published:** 2024-05-26

**Authors:** Ugur Canpolat

**Affiliations:** ^1^ Arrhythmia and Electrophysiology Unit, Department of Cardiology, Faculty of Medicine Hacettepe University Ankara Turkey

**Keywords:** atrial fibrillation, catheter ablation, fibrosis‐4 index, low‐voltage area, recurrence

In the current issue of the *Journal of Arrhythmia*, Yamada et al.^1^ retrospectively assessed the association of left atrial (LA) low‐voltage area (LVA) on electroanatomic mapping (bipolar voltage amplitude of <0.5 mV) with fibrosis index‐4 (FIB4), an indicator of liver fibrosis, during radiofrequency (RF)‐based first pulmonary vein isolation (PVI) at step 1 (*n* = 214) and tested the role of FIB4 ≥1.3 (cut‐off value for liver fibrosis) on atrial fibrillation (AF) recurrence after cryoballoon (CB)‐based first PVI (*n* = 129). The FIB‐4 index was strongly correlated with quantitative LA LVA (*r* = .642, *p* < .001). The FIB‐4 index ≥1.3 was also a significant predictor of the presence of LA LVA (OR: 2.508, *p* = .039) after adjusting for age, female gender, diabetes, non‐paroxysmal AF, and the LA diameter. Type IV collagen 7S, a marker of liver fibrosis, was also higher in the high FIB4 index group than in the low FIB4 index group. Post‐blanking period AF recurrence rate was 13.1% in the cryoablation group (20.2% in the high FIB4 group vs. 5.0% in the low FIB4 group, *p* = .017). The FIB‐4 index ≥1.3, an indirect indicator of LA LVA, is also an important predictor of AF recurrence in the cryoablation group (OR: 3.796, *p* = .037) after adjusting for the female gender, and non‐paroxysmal AF.

AF has a complex multifactorial pathophysiology. Several risk factors interplay a role in electrical, structural, and contractile atrial remodeling and subsequent occurrence and maintenance of AF. The latest consensus document recommended early catheter ablation for AF to prevent both the recurrence and progression of the disease and AF‐related hospitalizations. PVI is the cornerstone of all catheter ablation procedures in paroxysmal and persistent AF. The PVI can be achieved using various thermal and non‐thermal energy tools including RF, CB, laser balloon, hot balloon, and pulsed‐field ablation. Despite successful PVI, AF recurrence is still a significant problem. The atrial LVA (atrial substrate) plays a significant role in the recurrence of AF. However, there were conflicting data about the impact of atrial LVA ablation (substrate modification) in addition to PVI on AF recurrence after catheter ablation. Furthermore, there is a bidirectional relationship between atrial LVA and AF. Atrial LVA may be the cause and/or outcome of AF. A recent expert consensus statement on catheter and surgical ablation of AF recommended that ablation of atrial LVAs may only be reasonable during persistent AF ablation.^2^ Thus, non‐invasive pre‐procedural predictors of atrial LVA and/or AF recurrence are important in selecting the appropriate tool (CB or RF or others) and approach (PVI alone or PVI plus) for AF catheter ablation.

Metabolic dysfunction is a common pathophysiological pathway in various disease processes, particularly in heart‐liver interaction. Fatty liver diseases including non‐alcoholic fatty liver disease (NAFLD) and metabolic dysfunction‐associated fatty liver disease (MAFLD) are the most common etiology of chronic liver diseases and are considered as hepatic manifestations of the metabolic syndrome. Unlike NAFLD, the MAFLD diagnosis includes alcohol consumption and ≥1 metabolic syndrome criteria.^3^ Previous studies showed an association between MAFLD, liver fibrosis, and AF. However, the underlying mechanism causing liver fibrosis, atrial remodeling (atrial substrate or fibrosis), and AF is lacking. In a recent study, Decoin et al.^4^ demonstrated that high liver fibrosis scores [NAFLD Fibrosis Score] in MAFLD (fatty liver index >60+ the presence of metabolic comorbidities) patients are associated with adverse structural and contractile atrial remodeling (greater area of LA LVA on electroanatomical mapping, impaired LA reservoir function, greater LA volume = “atrial cardiomyopathy”) and AF recurrence following catheter ablation. Furthermore, the surgery subgroup of this study represented an increase in histopathological atrial fibrosis among patients with high fibrosis scores. Common pathophysiological pathways are proposed to be responsible for atrial and liver fibrosis. The fibroblast activation in the atrium and the hepatic stellate cell activation in the liver may act as a myofibroblast, causing an abnormal collagen formation. Immune cells including macrophages are critical in the current common pathways, mainly through transforming growth factor‐beta (TGF‐ß) and the nuclear factor kappa‐ß (NFKß) pathways. Contrarily, in a large‐scale population‐based study, van Kleef et al.^5^ demonstrated no association between abdominal ultrasound‐based fatty liver disease (NAFLD and MAFLD) and prevalent or incident AF. However, they proposed a different mechanism for liver stiffness (another marker of liver fibrosis) among AF patients in the absence of liver disease, which was proposed to be driven by venous congestion and congestive liver fibropathy. Thus, they suggested that AF patients with liver stiffness or fibrosis in the absence of apparent liver disease should be assessed for subclinical venous congestion. Therefore, the imaging of liver tissue for assessment of steatosis as a possible cause of stiffness and fibrosis is crucial.

The main criticism of biomarker‐based algorithms for assessing liver fibrosis is the lack of possibility for accurate adjustment analysis. The FIB‐4 index calculated in the study by Yamada et al.^1^ also has limitations for the prediction of atrial LVA and AF recurrence because the algorithm itself includes important predictors for AF and atrial remodeling, like age. Although the study population has had risk factors for fatty liver disease, the European Association for the Study of Liver Disease guideline on non‐invasive tests recommends transient elastography for screening of advanced liver disease among those with metabolic dysfunction and intermediate‐to‐high FIB‐4 index. In the absence of such a non‐invasive imaging test finding, it was impossible to be sure using the FIB‐4 index alone about the underlying mechanism of liver and atrial fibrosis in AF patients. Higher serum BNP levels in patients with higher FIB‐4 index may also be important for clarification of the underlying mechanism in the study by Yamada et al.^1^ Higher serum BNP levels may indicate diastolic dysfunction, heart failure with preserved ejection fraction, and subclinical venous congestion in the study. The higher FIB‐4 index as an indicator of liver fibrosis and atrial LVA may be caused by venous congestion and subsequent congestive hepatopathy rather than a fibrogenic mechanism in the study by Yamada et al.^1^ contrary to the proposed mechanisms. Therefore, the assessment of the liver using imaging tests (for steatosis and/or stiffness) and ventricular diastolic functions using detailed echocardiography may highlight the association of higher FIB‐4 index with atrial LVA and AF recurrence.

In conclusion, a higher FIB‐4 index as an indicator of liver and atrial fibrosis in the current study may be caused by AF itself alone or the bidirectional relation of fatty liver disease with AF. Comprehensive imaging of the liver and heart is crucial to reach an outcome. Without highlighting the underlying reason for the higher FIB‐4 index, we can not manage the patient appropriately. We should recommend assessing subclinical diastolic dysfunction and treatment in patients with liver fibrosis in the absence of apparent liver disease and AF. Furthermore, lifestyle modification and controlling of metabolic syndrome components in case of fatty liver disease‐related liver fibrosis and AF. The presence of liver and atrial fibrosis in both scenarios seems to be a marker (an outcome) rather than a cause of atrial LVA and AF recurrence.

## CONFLICT OF INTEREST STATEMENT

None declared.

## FUNDING INFORMATION

None declared.

## ETHICS STATEMENT

N/A.
